# Single-Component Hydrophilic Terpolymer Thin Film Systems for Imparting Surface Chemical Versatility on Various Substrates

**DOI:** 10.3390/polym16010044

**Published:** 2023-12-21

**Authors:** Yun Hee Ko, Hai Ha Tran Nguyen, Christopher R. Branstetter, Soeun Park, Jin-Kyun Lee, Jaesung Yang, Jangwook P. Jung, Myungwoong Kim

**Affiliations:** 1 Department of Chemistry and Chemical Engineering, Inha University, Incheon 22212, Republic of Korea; 12172006a@inha.edu (Y.H.K.); haiha.tran@inha.edu (H.H.T.N.); soeun2@inha.edu (S.P.); 2 Department of Applied Chemistry, Reutlingen University, Alteburgstraße 150, 72762 Reutlingen, Germany; 3 Department of Biological Engineering, Louisiana State University, Baton Rouge, LA 70803, USA; cbrans5@lsu.edu; 4 Program in Environment and Polymer Engineering, Inha University, Incheon 22212, Republic of Korea; jkl36@inha.ac.kr; 5 Department of Polymer Science and Engineering, Inha University, Incheon 22212, Republic of Korea; 6 Department of Chemistry, Yonsei University, Wonju 26493, Gangwon, Republic of Korea

**Keywords:** photocrosslinkable copolymer, functional pattern, functional composite, surface functionality, post-crosslinking functionalization

## Abstract

We demonstrate a single-component hydrophilic photocrosslinkable copolymer system that incorporates all critical functionalities into one chain. This design allows for the creation of uniform functional organic coatings on a variety of substrates. The copolymers were composed of a poly(ethylene oxide)-containing monomer, a monomer that can release a primary amine upon UV light, and a monomer with reactive epoxide or cyclic dithiocarbonate with a primary amine. These copolymers are easily incorporated into the solution-casting process using polar solvents. Furthermore, the resulting coating can be readily stabilized through UV light-induced crosslinking, providing an advantage for controlling the surface properties of various substrates. The photocrosslinking capability further enables us to photolithographically define stable polymer domains in a desirable region. The resulting copolymer coatings were chemically versatile in immobilizing complex molecules by (i) post-crosslinking functionalization with the reactive groups on the surface and (ii) the formation of a composite coating by mixing varying amounts of a protein of interest, i.e., fish skin gelatin, which can form a uniform dual crosslinked network. The number of functionalization sites in a thin film could be controlled by tuning the composition of the copolymers. In photocrosslinking and subsequent functionalizations, we assessed the reactivity of the epoxide and cyclic dithiocarbonate with the generated primary amine. Moreover, the orthogonality of the possible reactions of the presented reactive functionalities in the crosslinked thin films with complex molecules is assessed. The resulting copolymer coatings were further utilized to define a hydrophobic surface or an active surface for the adhesion of biological objects.

## 1. Introduction

With the increase in demand for high-performance and more complex materials, the importance of surface engineering has also been highlighted. Therefore, modifying the surfaces of different substrates to attain the desired structures and properties for specific applications has attracted considerable interest in the field of materials science [1,2]. The surface of a material is a point of interaction with the external environment and other materials [3,4]. Many issues associated with complex systems consisting of different materials can be solved by understanding the physical and chemical interactions that occur at the surfaces or the interfaces of the material. Therefore, controlling the properties of such surfaces and interfaces is critical. An effective way to modify surfaces for improving or tuning properties, such as wettability [5,6,7], adhesion [8,9,10], roughness [11], polarity [12], and reactivity [13,14], is through the deposition of a functional polymer, in which the desired surface functionalities can be conferred by controlling the architecture through the design of the chemical structure at a molecular level. Moreover, such a deposition offers high processability with appropriate solvents and stability under a careful design to include reactive moieties. Examples include a conductive polymer coating to improve the performance of lithium–sulfur batteries [15,16] and organic thin films to provide chemical functionalities for improving the adhesion of biological objects in aqueous media [17,18].

However, precise control of the surface chemistry is still challenging. For instance, one of the most common methods to do so is exposure to UV light/ozone or plasma [19,20]. While such approaches are simple and effective, the chemical functionalities are not well preserved, thus leading to significant deterioration of the polymer structure [21,22,23]. Therefore, it is important to design polymers that can overcome these challenges at a molecular level [24,25,26]. The design guidelines can be followed for the polymers suitable for surface modification. First, the polymer thin film should have functionalities that can impart stability against an external environment, such as a solvent, in a controlled manner. Second, the polymer should be processable in a certain way, such as by being cast in the form of a solution onto the target surface. Third, the polymer should have components that can impart core properties for desirable applications. These components include reactive chemical functionalities that can be utilized to further incorporate pivotal functionalities [27] as well as to form crosslinked networks [28].

Recently, it was reported that stable thin films can be formed using copolymers with a photoactivatable *o*-nitrobenzyl derivative, which has effective photosensitive functionalities [29,30], a reactive epoxide [31], or cyclic dithiocarbonate [32]. The released amine reacts with another reactive group to form a stable thin film. This copolymer platform was not only beneficial in fabricating a stable thin film, but it also showed potential applicability in a universal coating system, exhibiting substrate independence. However, the system consists of at least two components: (i) a copolymer or a crosslinker with multiple photocleavable units to yield a nucleophile, and (ii) a copolymer or a crosslinker bearing reactive moieties that can react with the generated nucleophile to form the crosslinked network. Many components in polymer systems typically exhibit limited compatibility, even though the components are structurally similar, due to the nature of the polymer [33]. The application of multi-component systems is highly limited for composite materials, as when the structurally dissimilar complex polymer is mixed, it generally results in phase separation [34], which is not desired for a thin film. In addition, the incompatibility makes it difficult to thoroughly understand the crosslinking reactions. Thus, as an ideal system to address these issues, a single-component copolymer that bears all functionalities in one chain needs to be achieved. Although many types of polymeric systems, including simple epoxy-, acrylate-, or azlactone-based monomers and copolymers, have been reported [17,35,36,37,38], it is a challenging task, in the single-component polymer, to simultaneously achieve controlled reactivities for crosslinking on many different types of substrates and post-crosslinking surface functionalization under mild conditions in predictable and appreciable manners. Apparently, chemical routes to realize a rationally designed single-component copolymer system satisfying the above-mentioned criteria should lead us to universal thin film systems based on organic/polymeric materials exhibiting multiple chemical functionalities.

In this work, we attempted a terpolymer thin film system containing poly(ethylene glycol) methyl ether methacrylate (PEGMEMA), glycidyl methacrylate (GMA), or cyclic dithiocarbonate methyl methacrylate (DTCMMA), and 2-((((2-nitrobenzyl)oxy)carbonyl)amino)ethyl methacrylate (NBOCAEMA) by reversible addition-fragmentation chain transfer (RAFT) polymerization to yield poly(PEGMEMA-*r*-GMA-*r*-NBOCAEMA) (MGN) or poly(PEGMEMA-*r*-DTCMMA-*r*-NBOCAEMA) (MDN). PEGMEMA was the major component that imparted hydrophilicity for casting with a polar solvent to achieve processability on organic and inorganic substrates. The composition of the copolymer was varied to control the amounts of reactive groups such as a primary amine, an epoxide, or a DTC group. The photocrosslinking capability and photopatternability of the copolymers to form stable thin films and patterns were assessed. The reactivity and orthogonality of the functional groups in the thin films with complex molecules, such as fluorescent dye molecules and a protein of interest, upon crosslinking were also investigated. The resulting thin films were further used to fabricate a hydrophobic surface and a well-defined and uniform composite thin film using fish skin gelatin.

## 2. Materials and Methods

### 2.1. Materials

2-((((2-Nitrobenzyl)oxy)carbonyl)amino)ethyl methacrylate (NBOCAEMA), cyclic dithiocarbonate methylene methacrylate (DTCMMA), 4-cyano-4-[(dodecylsulfanylthiocarbonyl)sulfanyl]pentanoic acid (CDSTSP), and methacrylate-incorporated fish skin gelatin (fGelMA; the degree of the addition reaction = 0.920) were synthesized through previously reported procedures [35]. Potassium *tert*-butoxide (97%), 2-nitrobenzyl alcohol, 1-dodecylamine (>98%), and lithium bromide (>99.0%) were obtained from Alfa Aesar (Ward Hill, MA, USA). Dibutyltin dilaurate (DBTL), fluorescein isothiocyanate isomer I (FITC; >90%), hexylamine (>99%), and 2-isocyanatoethyl methacrylate were purchased from TCI Chemicals Co., Ltd. (Tokyo, Japan) Heptane (>98.0%). Sodium sulfate (Na_2_SO_4_; >99.0%), ethyl acetate (99.5%), *n*-hexane (>95.0%), acetonitrile (>99.5%), dimethyl sulfoxide (DMSO; 99.5%), and tetrahydrofuran (THF) were purchased from Daejung Chemicals & Metals Co., Ltd. (Siheung, Korea) Phosphate-buffered saline (PBS; 10× solution, pH = 7.4 ± 0.1), dichloromethane (DCM; 99.5%), diethyl ether (99.0%), and ethanol (94.5%) were obtained from Samchun Chemicals (Seoul, Korea). Furthermore, 2,2′-azobis(2-methylpropionitrile) (AIBN) was purchased from Junsei Chemicals Co., Ltd. (Tokyo, Japan) and recrystallized from methanol before use. Tetramethylrhodamine (TAMRA) amine (5-isomer) and 3-cyanine 5 maleimide (Cy5 maleimide) were supplied by Lumiprobe (Wan Chai, Hong Kong). Poly(ethylene glycol) methyl ether methacrylate (PEGMEMA; 99%, average M_n_ = 300 g/mol), 1,4-dioxane (anhydrous, 99.8%), lithium phenyl-2,4,6-tirmethyl-benzoylphosphinate (LAP; >95%), bovine serum albumin (BSA; >95%), glycidyl methacrylate (GMA; >97%), 2,2-dimethoxy-2-phenylacetophenone (DMPA; 99%), and albumin–fluorescein isothiocyanate conjugate (BSA-FITC) were purchased from Sigma-Aldrich Co., Ltd. (Saint Louis, MO USA) 1*H*,1*H*,2*H*,2*H*-perfluorooctyl methacrylate (FOMA) was obtained from Shanghai Xinglu Chemical Technology Co. (Shanghai, Chaina) PEGMEMA and GMA were purified by passing them through a neutral alumina column to remove the inhibitor prior to polymerization. To obtain the cultures of the C2C12 myoblasts (ATCC, Manassas, VA, USA, CRL-1772), fetal bovine serum (Thermo Fisher Scientific, Waltham, MA, USA, MT35010CV, USDA tested), and *L*-glutamine (Thermo Fisher Scientific, Waltham, MA USA, 25-005-CI), Penicillin/Streptomycin (P/S, Lonza, Walkersville, MD, USA, 17-602E) were added to the DMEM (Dulbecco’s Modified Eagle’s Medium, Thermo Fisher Scientific, Waltham, MA, USA, 10-013-CV). To subculture, TrypLE (Thermo Fisher Scientific, Waltham, MA, USA, 12604013) was used. Prior to culture initiation, the composite films were sterilized with gentamicin (Thermo Fisher Scientific, Waltham, MA USA, 15750060). To assess the adhesion of C2C12 myoblasts to thin films, 4% paraformaldehyde (PFA, Electron Microscopy Sciences, Hatfield, PA USA, 15710), Triton-X 100 (Sigma-Aldrich, Saint Louis, MO, USA, X-100), PBS (Thermo Fisher Scientific, Waltham, MA, USA, BP399), phalloidin (Biotium, Fremont, CA USA, 00041), and Hoechst 33342 (AnaSpec, Fremont, CA, USA, AS83218) were used.

### 2.2. General Copolymerization

Desired amounts of PEGMEMA, DTCMMA (or GMA), NBOCAEMA, CDSTSP, AIBN, and 1,4-dioxane were added and mixed in a Schlenk flask, followed by degassing with three freeze–pump–thaw cycles. The feed compositions for MGN and MDN are tabulated in Table 1. The ratio of mole concentrations of the reactants, i.e., [monomers]:[CDSTSP]:[AIBN], was set to 79:1:0.5, and the concentration of solvent was 50 wt%. The copolymerization was performed at 70 °C for 4 h, then quenched by cooling down to room temperature and subsequent exposure to air. The reaction solution was diluted with THF and added dropwise to diethyl ether (for MDN) or *n*-hexane (for MGN). The supernatant was discarded, and the resultant yellow solid was collected and dried under a vacuum overnight to obtain the final polymer product.

### 2.3. Photocrosslinking of Thin Films

A certain amount of MDN or MGN random copolymer was dissolved in ethyl alcohol to prepare a 2.5 wt% solution. Prior to spin-coating, the solution was filtered through a syringe filter (pore size = 0.45 μm). A silicon wafer (1 cm × 1 cm) was thoroughly washed with acetone, isopropyl alcohol, and toluene and dried under a nitrogen stream. The prepared solution was spin-coated on the cleaned wafer at 4000 rpm for 30 s. The film was exposed to UV light (λ = 254 nm) at an intensity range of 0–500 mJ/cm^2^, followed by placing the sample at room temperature for 10 min and subsequent development with deionized (DI) water for 8 min. For the pattern formation, the spin-coated sample with MGN or MDN was exposed to UV light (λ = 254 nm) with an energy of 500 mJ/cm^2^ through a photomask with the following pattern scale: 18 µm/18 µm line/space and 20 µm × 20 µm square. Thereafter, the sample was kept at room temperature for 10 min, then immersed in DI water for 8 min, and dried under a nitrogen stream. For stability testing, the MGN or MDN film was immersed in toluene or DI water for 1–3 days or was repetitively sonicated in DI water for a minute.

### 2.4. Post-Crosslinking Immobilization of Complex Molecules

The MDN (2.5 wt%) or MGN (2.5 wt%) in ethyl alcohol was spin-coated at 4000 rpm for 30 s on a silicon substrate. UV light was illuminated at an intensity of 500 mJ/cm^2^ through a photomask. Thereafter, the film was placed at room temperature for 10 min and then developed in DI water for 8 min. For the immobilization of TAMRA or FITC, the resulting MDN or MGN pattern sample was immersed in a solution of TAMRA in toluene (0.15 wt%) or in the FITC solution (5.0 mg in a mixed solution of 0.2 mL of DMSO and 10 mL of PBS) for 1 h, followed by washing with DI water. In the case of the immobilization of the Cy5 maleimide, the MDN sample was dipped in the Cy5 maleimide solution (5.0 mg in a mixed solution of 0.2 mL of DMSO and 10 mL of PBS) for 1 h, followed by washing with DI water. For immobilization of BSA-FITC, the MGN or MDN pattern sample was immersed in an aqueous solution of BSA-FITC (BSA-FITC 5 mg in 9 mL of DI water + 1 mL of PBS) for 1 h, followed by washing with DI water. For the surface modification with nonpolar molecules, photocrosslinked MGN1 or MDN1 thin film was immersed in a solution of hexylamine or dodecylamine in toluene (20 wt%) for 1 h. FOMA was incorporated into the MDN5 thin film by dipping in solutions of FOMA (20 wt%) and DMPA (5 wt% relative to FOMA) in toluene and subsequent UV light illumination (λ = 365 nm; UVlink CL-508 Crosslinker) for 1 h.

### 2.5. Formation of Photocrosslinked Composite Thin Films with fGelMA

Here, 20 mg of MGN or MDN was mixed with fGelMA (10, 50, or 100 wt% of MGN or MDN), LAP (0.05 wt%), and DI water to achieve a 2.5 wt% concentration. The spin-coating of the filtered sample with the syringe filter was conducted onto a Si or polystyrene (PS) substrate at 4000 rpm for 30 s, followed by the UV light illumination at 500 mJ/cm^2^. Then, the sample was placed at room temperature for 10 min and immersed in DI water for 8 min. The incorporated fGelMA was labeled with FITC in the same procedure as described above.

### 2.6. Characterization

The ^1^H NMR spectra were recorded on a JNM-ECZ400S spectrometer (JEOL) in CDCl_3_ or DMSO-*d*_6_. The molecular weight information of the synthesized copolymers was acquired with size exclusion chromatography (SEC) using a Thermo Scientific Ultimate 3000 system (eluent: THF; flow rate = 1 mL/min; column temperature = 35 °C). Ten PS standard samples (M_n_ range: 1.2–2700 kg/mol) were utilized for constructing a calibration curve. The thickness was determined using an SE MG-1000 spectroscopic ellipsometer (Nano-View Co., Ltd.) in the wavelength range of 350–840 nm. The thickness was estimated by fitting the ellipsometric parameters (Ψ and Δ) measured at an angle of 70° with the Cauchy layer model. Fluorescent microscopic images were obtained using a 510 META confocal laser scanning microscope (CLSM; Zeiss). Excitation wavelengths for FITC, TAMRA, and Cy5 dyes are 488, 543, and 633 nm, respectively. X-ray photoelectron spectroscopy (XPS) was conducted using Thermo Scientific K-Alpha. The water contact angle was measured using a contact angle analyzer (SEO, Phoenix-MT).

### 2.7. Adhesion and Growth of C2C12 Myoblasts

To assess the growth of the C2C12 myocytes on each thin film, composite thin films with fGelMA were taped to the bottom of a well in a 12-well plate using double-sided tape to prevent the surfaces from floating, followed by sterilization with 50 µg/mL gentamicin for 15 min. Next, the C2C12 myoblasts were seeded at a density of 10,000 cells/cm^2^, cultured for 24 h at 37 °C/5% CO_2_, fixed with 4% PFA for 15 min, washed three times with PBS, permeabilized with 0.1% Triton-X 100 for 10 min, and washed three times with PBS again. To visualize the cell attachment on the composite thin film, the C2C12 myoblasts were stained with phalloidin (10 µM), following the manufacturer’s protocol, and counterstained with Hoechst 33342. Using the Nikon Ti2 inverted microscope equipped with a pco.edge 4.2 camera, all the images were taken with a 20× objective lens (NA = 0.45) at an exposure time of 250 ms. The Hoechst images were processed using FIJI software (2.9.0) by automatically adjusting the image threshold with the black and white settings, converting the image to a binary mask, and applying a binary watershed filter to separate the adjacent cells. The number of cells was counted using the analysis particle program with size settings of 120 pixel^2^–infinity, ignoring cells along the edge of the images. The phalloidin images were analyzed by automatically adjusting the brightness and contrast of the image and then automatically adjusting the threshold with the black and white settings. Similar to the Hoechst 33342-stained images, the phalloidin images were converted to a binary mask, with the total area and area fraction measured using the FIJI software.

## 3. Results and Discussion

### 3.1. Synthesis of Hydrophilic Terpolymers by RAFT Copolymerization

**Scheme 1:**
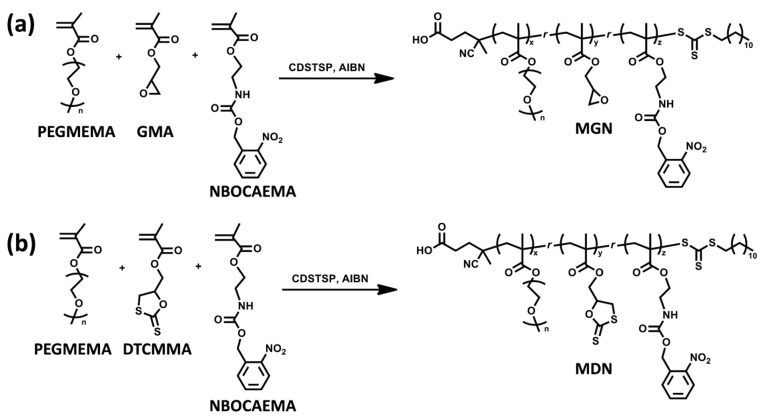
RAFT copolymerization of PEGMEMA, NBOCAEMA, and (**a**) GMA or (**b**) DTCMMA.

A series of MGN and MDN copolymers were synthesized by RAFT copolymerization (Scheme 1). The resulting copolymers exhibited M_n_ values in the range of 13–23 kg/mol, which is close to the M_n_ expected from the ratio of CDSTSP and monomers (Table 1). Moreover, *Ð* was observed to be in the range of 1.26–1.53 (Table 1) with a typical unimodal shape (Figures S1 and S2), which is the typical molecular weight distribution observed from the polymers synthesized with a control under a reversible deactivation radical polymerization mechanism, regardless of the variations in the feed composition [39,40]. In the ^1^H NMR spectra of all the MGN and MDN samples, all the characteristic peaks of PEGMEMA, NBOCAEMA, and GMA or DTCMMA were observed. Specifically, the peak at ~3.2 ppm (assigned to three protons of the end methyl group of PEG in PEGMEMA), the peak at ~8.1 ppm (assigned to a proton of the urethane group in NBOCAEMA), the peak at ~2.8 ppm (assigned to protons of CH_2_ of the epoxide group in GMA for MGN), or the peak at ~5.5 ppm (assigned to a proton of CH in DTCMMA) were utilized to estimate the actual compositions. As shown in Table 1, the actual compositions of MGN and MDN copolymers are not dissimilar from their feed compositions: when the feeding amount of NBOCAEMA was higher, lower, or equal to that of GMA (or DTCMMA), the actual compositions were also following the same trend. Therefore, by simply tuning the copolymer composition, it is feasible to control the relative amount of each surface reactive functionality originating from the type of monomer (i.e., primary amine from NBOC, DTC from DTCMMA, thiol from DTCMMA, and epoxide from GMA) of the photocrosslinked thin films fabricated from these copolymers.

### 3.2. Photocrosslinking of Thin Films and Photopatternability

**Scheme 2:**
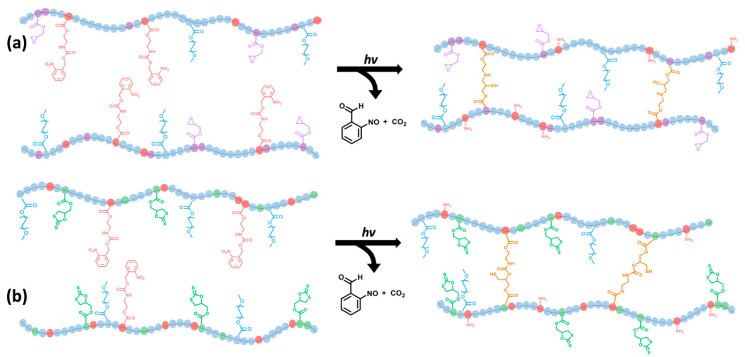
Photocrosslinking process in the (**a**) MGN and (**b**) MDN thin films.

**Figure 1:**
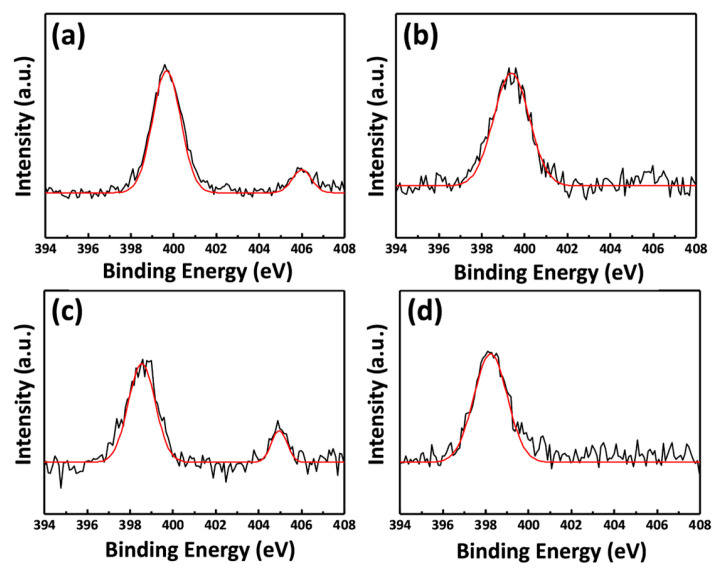
Multiplex N_1s_ XPS spectra of the MGN5 thin film (**a**) before and (**b**) after photocrosslinking, and the MDN5 thin film (**c**) before and (**d**) after photocrosslinking.

Scheme 2 illustrates the mechanism of the photocrosslinking processes in the MGN and MDN thin films under UV light illumination. The NBOC group absorbs the illuminated light to undergo a photocleavage reaction, releasing a pendant primary amine, free *o*-nitrosobenzaldehyde, and carbon dioxide. The released primary amine attacks the epoxide or the DTC on other chains to form the crosslinked network. The thin films made from MGN5 and MDN5 were subjected to XPS measurements; upon UV exposure and subsequent washing, the peak at ~406 eV assigned to nitrogen in the NO_2_ group disappeared in the XPS spectra (Figure 1) [41,42], thereby confirming that the cleavage reaction occurred and the residual byproducts were removed.

**Figure 2:**
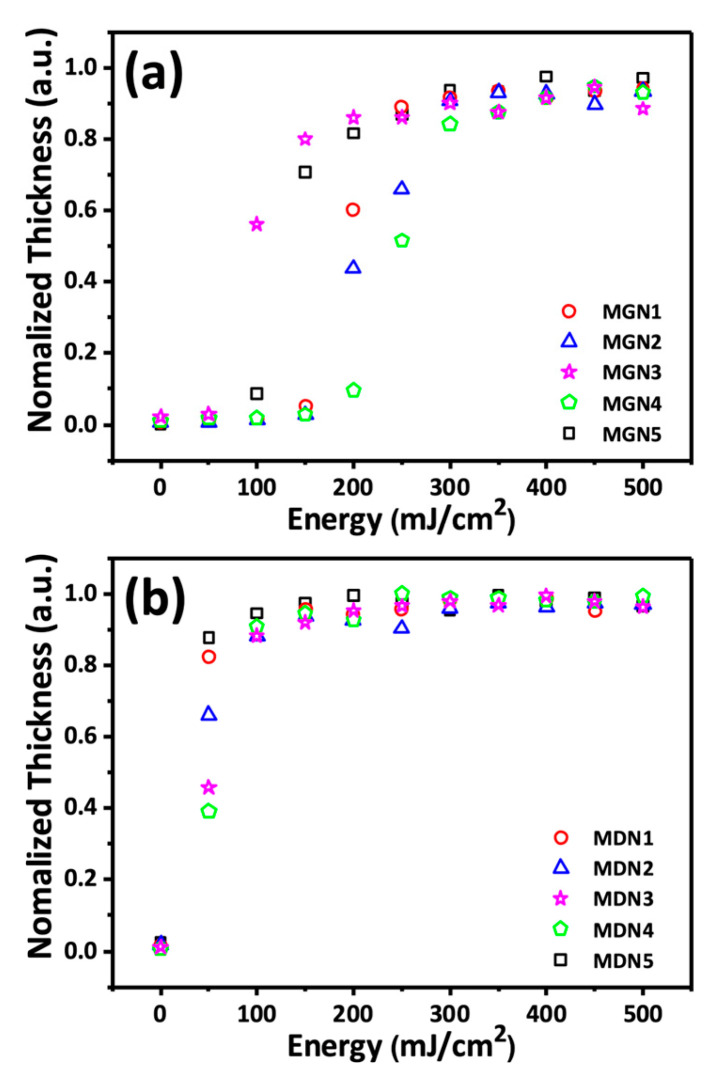
Normalized thickness as a function of the energy of the UV light used for illuminating the thin films of (**a**) MGN and (**b**) MDN.

Then, the photocrosslinking behaviors for both MGN and MDN were assessed by measuring the thickness of the thin films fabricated with varying light energies. Approximately 60 nm-thick films were fabricated by spin-coating the solutions of MGN or MDN copolymers, followed by the illumination of UV light with an energy of 0 to 500 mJ/cm^2^ at intervals of 50 mJ/cm^2^. Then, the film was immersed in water for 8 min. Figure 2 shows the thickness normalized to the thickness of the thin films before immersion in DI water as a function of the energy of the UV light used for the illumination. All the MGN and MDN films exhibited full crosslinking at values higher than 300 mJ/cm^2^, regardless of the composition of the copolymer. The MDN copolymer thin films required ~100 mJ/cm^2^ to attain the normalized thickness of 0.90 (*E*_90_) for all the compositions (Figure 2b); however, *E*_90_ for all the MGN thin films was three times higher than that for the MDN thin films (Figure 2a). Since this is the case for the copolymers at the same composition, e.g., MDN3 and MGN3, this significant difference between the two is likely due to the type of the reactive ring group and hence the difference in the reactivity between the DTC and epoxide. In this system, the DTC reacts more efficiently with the pendant primary amine than the epoxide, resulting in better sensitivity to UV light. The required energy for the normalized thickness of 0.50 (*E*_50_) was found to largely vary due to the composition of the MGN copolymer; however, *E*_50_ for the MDN copolymers does not, further suggesting that the reactivity of the DTC differs from that of the epoxide in the thin film geometry. The variation in *E*_50_ for the MGN copolymers tends to relate to the number of reactive groups (*F*_NBOC_ and *F*_GMA_). The MGN3 thin film was the most sensitive to UV light, with an *E*_50_ of ~100 mJ/cm^2^. The *E*_50_ values of MGN1 and MGN5, which have highly unbalanced compositions between the NBOC and the GMA groups, were in the range of 130–170 mJ/cm^2^. In contrast, the *E*_50_ values of MGN2 and MGN4, where the degrees of composition unbalance are lower than those of MGN1 and MGN5, were in the range of 210–250 mJ/cm^2^. These results suggest that the concentration of the reactive groups controls the crosslinking reaction, and therefore the sensitivity for photocrosslinking is dependent on the composition of the MGN. Moreover, the reactivity of the epoxy group is lower than that of the DTC group with the primary amine group in the crosslinking reaction in the solid state, highlighting the importance of the type of reactive group for the crosslinking process.

**Figure 3:**
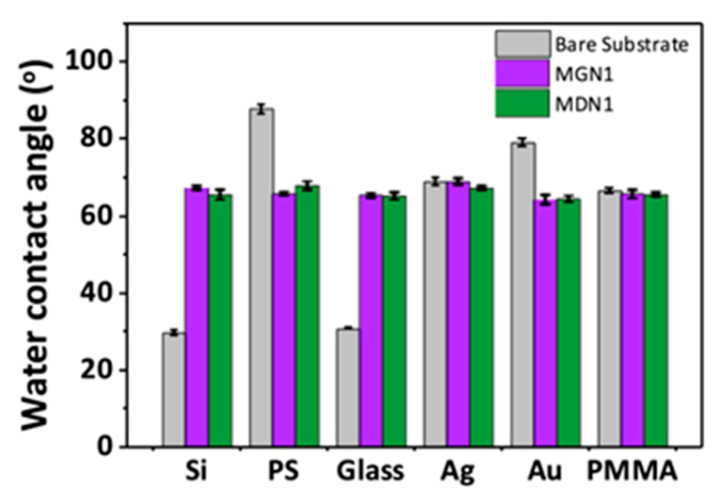
Water contact angles of bare, MGN1-coated, and MDN1-coated surfaces.

The process for the thin film deposition was performed with the copolymer solution in ethanol, which typically does not chemically damage organic and inorganic substrates. Both MGN1 and MDN1 were spin-coated onto various substrates, including silicon, PS, glass, Ag, Au, and PMMA, and photocrosslinked at 500 mJ/cm^2^. The water contact angles measured on the bare substrates largely varied from 20° to 90°; however, the contact angle values after the coating of MGN1 and MDN1 were all comparable, regardless of the substrate type (Figure 3 and Figure S3). These results confirm that the photocrosslinkable copolymers are processable on different substrates, highlighting their potential and applicability toward universal coating materials.

The stability of the fabricated thin films was further assessed by immersion in DI water and toluene. The thickness and the water contact angle of MDN3 and MGN3 thin films were not changed after immersion for up to three days (Figure S4a,b), confirming that the thin films are stable enough that they can be utilized for further modification reactions and applications. Next, the thin films were subjected to repetitive sonication processes in DI water and toluene. Both MDN3 and MGN3 thin films did not show significant changes in the thickness or the water contact angle (Figure S4c,d), suggesting that the crosslinking chemistry provides the route to attaining stable polymeric thin film coatings.

**Figure 4:**
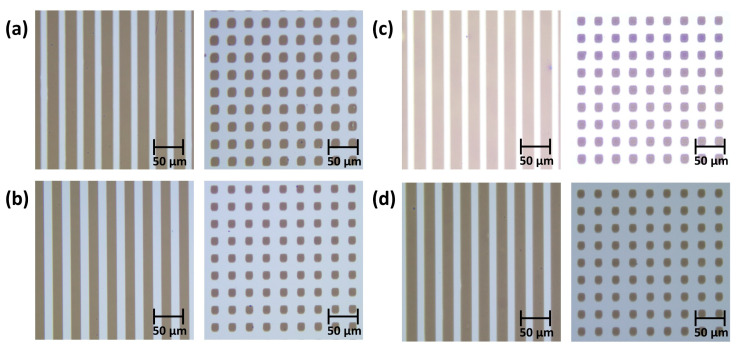
Representative optical micrographs showing line/space and square patterns of (**a**) MGN1, (**b**) MGN5, (**c**) MDN1, and (**d**) MDN5 (scale bar = 50 µm).

Since the MGN and MDN copolymers showed photocrosslinking capability in a thin film, we further attempted to define patterns in a photolithographic manner. A photomask with a line/space (18 μm/18 μm) pattern and a square pattern (20 μm × 20 μm, distance between squares = 25 μm) was placed in contact with the copolymer thin film. Subsequently, UV light was illuminated through the photomask at 500 mJ/cm^2^, followed by the same post-exposure process. As shown in Figure 4 and Figure S5, the patterns in the photomask were effectively transferred to all the MGN and MDN copolymer thin films, which resulted in negative-tone photopatterns. Even with the simple lithographic setup, the minimum critical dimension of ~3 μm was attainable with both MGN3 and MDN3 copolymers (Figure S6). Since the *o*-nitrobenzyl group is known to be capable of a cleavage reaction with an electron beam as well as UV light at a wavelength of ~250 nm or less [43,44], the chemical designs will enable resolution at the sub-micron scale using either deep UV or electron beam lithographic techniques. These results highlight that the copolymer system can be utilized to define patterns with specific chemical functionalities in a desired region.

### 3.3. Post-Crosslinking Surface Functionalization Reactions with Preserved Chemical Functionalities

**Figure 5:**
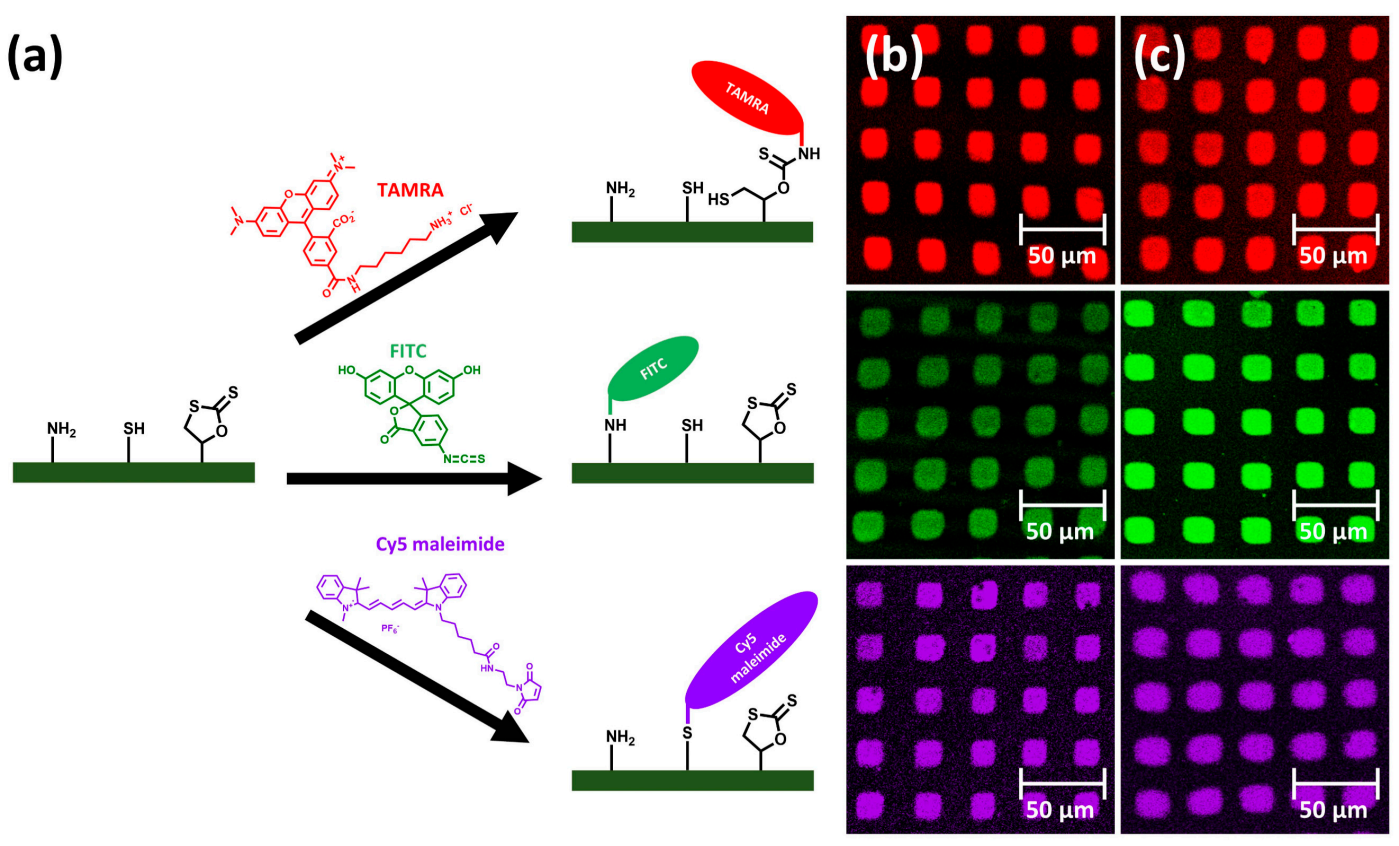
(**a**) Functionalization routes of the three fluorescent dye molecules with functional groups that can react with the chemical functionalities in the MDN copolymer thin film and square patterns functionalized with the TAMRA amine (**top**), FITC (**middle**), and Cy5 maleimide (**bottom**) in the (**b**) MDN1 and (**c**) MDN5 thin films (scale bar = 50 µm).

**Figure 6:**
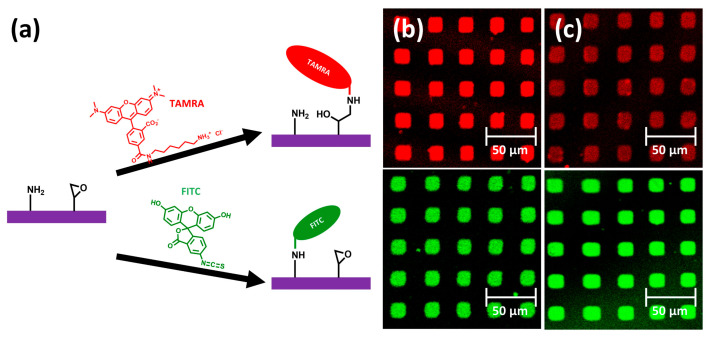
(**a**) Functionalization routes of two fluorescent dye molecules having functional groups that can react with the chemical functionalities in the MGN copolymer thin film and square patterns functionalized with TAMRA amine (**top**) and FITC (**bottom**) in (**b**) MGN1 and (**c**) MGN5 thin films (scale bar = 50 µm).

Upon photocrosslinking, the thin films should have different reactive functionalities. In the MDN thin films, three functionalities are present: unreacted DTC, primary amine, and thiol released after the ring-opening reaction of DTC with the amine (Figure 5a). To assess if these functionalities are available for post-crosslinking functionalization, three types of fluorescent dye molecules bearing different reactive groups were utilized, namely TAMRA amine, FITC, and Cy5 maleimide, bearing a primary amine (reactive with the surface DTC group), isothiocyanate (reactive with the surface primary amine group), and maleimide (reactive with surface thiol), respectively [31,45,46]. For the dye incorporation reactions, we first prepared square patterns of MDN1 (*F*_DTC_ > *F*_NBOC_; DTC-rich) and MDN5 (*F*_DTC_ < *F*_NBOC_; primary amine-rich). Each dye molecule was incorporated by a simple immersion of the pattern sample into the solution of the dye. After the functionalization, in the CLSM images taken from the MDN1 and MDN5 samples (Figure 5b,c), all three dyes were successfully detected on the square patterns at appropriate excitation and observation wavelengths. Moreover, the functionalization of FITC onto the photopattern with the minimum critical dimension of ~3 μm was successfully achieved (Figure S6). These results suggest that in the terpolymer system, although portions of two reactive groups are consumed, the unreacted functionalities are present and available for defining specific chemical functionalities. Interestingly, the fluorescence intensities of TAMRA and FITC varied according to the type of thin film. The primary amine-rich MDN5 thin film exhibited a stronger fluorescence signal from FITC than the DTC-rich MDN1 thin film (Figure S7). In the case of TAMRA, reverse trends were observed: a slightly stronger fluorescence signal was observed from MDN1 than that from MDN5. For MGN1 and MGN5, two reactive functionalities in the crosslinked thin films, primary amine and unreacted epoxide, were subjected to reactions with the TAMRA amine and FITC (Figure 6a). Both MGN1 and MGN5 also showed effective functionalization with the two dye molecules (Figure 6b,c). Along with the results of the MDN series, the epoxide-rich MGN1 also showed a stronger fluorescence signal from TAMRA than MGN5, and the amine-rich MGN5 exhibited a higher fluorescence intensity from FITC than MGN1 (Figure S7). These results strongly suggest that the concentrations of the specific reactive groups can be effectively varied by tuning the composition. It is noted that the slight difference in the intensities of the fluorescence from TAMRA in MDN1 and MDN5 is dissimilar from the behaviors of MGN1 and MGN5. This is likely due to the higher reactivity of the DTC ring than the epoxide ring: a larger amount of the DTC ring than the epoxide ring participates in the crosslinking reaction, which is in good agreement with the photocrosslinking studies. Thus, the concentration of the DTC ring is lower than expected, leading to a low concentration of TAMRA functionalized by the reaction with the DTC ring and, hence, a relatively low fluorescence intensity.

**Figure 7:**
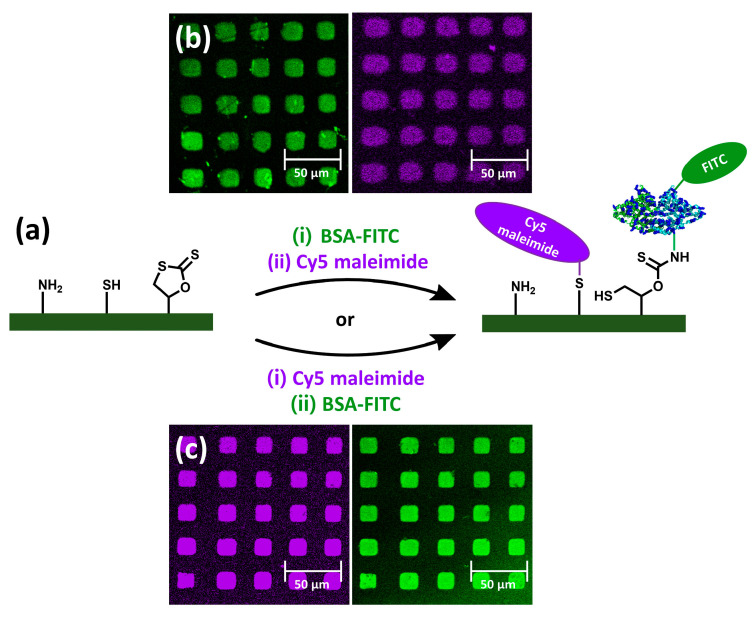
(**a**) Two sequential functionalization routes of BSA-FITC and Cy5 maleimide, and (**b**,**c**) CLSM images showing the fluorescent signal from the square patterns of the MDN5 copolymer thin films where BSA-FITC and Cy5 were incorporated through two different routes (scale bar = 50 µm).

The presence of various reactive groups in the copolymer thin films enables the definition of multiple chemical functionalities by post-crosslinking modification reactions. To assess the feasibility of attaining multifunctional surfaces, two distinctive molecules, i.e., BSA-FITC and Cy5 maleimide, were functionalized onto the MDN5 thin films (Figure 7). Two routes were tested to confirm that the two reactions do not interfere with each other: (i) an immersion in the solution of BSA-FITC and a subsequent immersion in the solution of Cy5 maleimide, and (ii) an immersion in the solution of Cy5 maleimide and a subsequent immersion in the solution of BSA-FITC. The CLSM images obtained from the samples treated by the two routes showed that both molecules were effectively incorporated in the MDN5 thin films, regardless of the order of the incorporation reaction. These results confirm that the DTC or thiol remain almost intact during the functionalization of BSA-FITC or Cy5 maleimide, strongly suggesting the feasibility of the orthogonal functionalization of the two different molecules on the surface.

**Figure 8:**
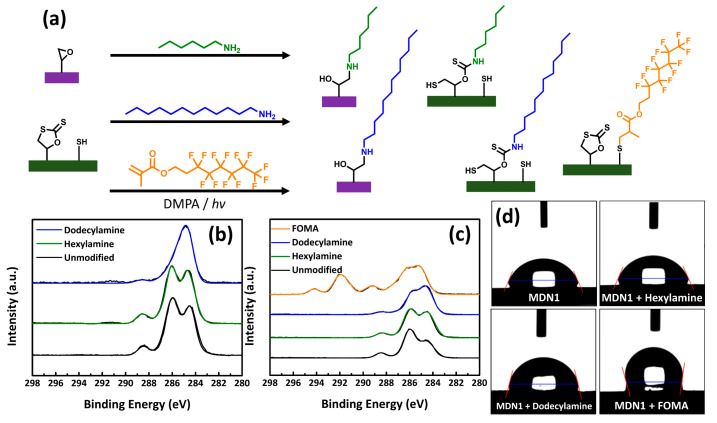
(**a**) Scheme showing immobilization of various nonpolar alkyl amines and methacrylate on MGN and MDN thin films. XPS C1s multiplex spectra of (**b**) unmodified, hexylamine-, and dodecylamine-immobilized MGN1 thin films, and (**c**) unmodified, hexylamine-, dodecylamine-, and FOMA-immobilized MDN1 thin films. (**d**) Optical images showing water sessile drops on the surfaces of unmodified, hexylamine-, dodecylamine-, and FOMA-immobilized MDN1 thin films.

The post-crosslinking modification strategy was further employed to control the surface wetting behavior. We utilized two chemical routes to attain more hydrophobic surfaces: (i) the addition reaction of hexylamine or dodecylamine to the epoxide or DTC ring in the MGN1 or MDN1 thin film, and (ii) the light-mediated thiol-ene click reaction of FOMA with the surface thiol group of the MDN1 thin film (Figure 8a). All the reactions were conducted under mild conditions in simple manners. The addition reactions with the primary amine compounds were performed by simply immersing the sample into amine solutions in toluene at room temperature. The thiol-ene reaction was performed by UV illumination of the sample in the solutions of FOMA and DMPA in toluene at room temperature. The modified thin films were subjected to XPS measurements to confirm the immobilized reactions (Figure 8b,c). The unmodified MGN1 and MDN1 thin films showed characteristic peaks at ~284 eV (carbon in C-C), ~286 eV (carbon in C-O and C-N), and ~288.5 eV (carbon in O-C=O) [47,48]. The incorporation of hexylamine into the thin films of MGN1 and MDN1 led to a slight increase in the peaks at ~284 eV. Upon addition of dodecylamine, the intensities of the peaks at ~284 eV for both MGN1 and MDN1 increased dramatically (blue traces), indicating that the surfaces were covered by alkyl chains. After the thiol-ene reaction of the MDN1 thin film with FOMA, the peaks emerged at ~292 eV and ~294 eV, which are assigned to carbons in CF_2_ and CF_3_, respectively [47], confirming the successful immobilization by the click reaction. Since the surfaces were modified with nonpolar molecules, the wetting characteristics were expected to be largely changed. For both MGN1 and MDN1, the immersion in the solution of hexylamine in toluene led to a slight increase (~3°) in the water contact angle from 67.2 ± 0.5° and 65.5 ± 1.3° to 69.8 ± 1.4° and 68.1 ± 0.8°, respectively (Figure 8d and Figure S8), which is in good agreement with the slight increase in the C-C peak in the XPS studies. After the treatment with dodecylamine, the water contact angles increased by ~10° (78.6 ± 1.2° and 78.1 ± 1.5° for MGN1 and MDN1, respectively), as the incorporation of the molecule having a longer alkyl chain expectedly exhibits a greater increase in water contact angle. In addition, the thiol group in the MDN1 thin film was exploited to immobilize FOMA by the thiol-ene click reaction undergone by active radical species generated from DMPA after UV light illumination. After the reaction, the water contact angle increased dramatically by approximately 40° (102.4 ± 1.5°), which was expected because of the exceptionally hydrophobic property of perfluorinated copolymers [49].

### 3.4. Fabrication of Composite Thin Films with fGelMA to Attain Biologically Active Surfaces

The terpolymer systems can readily form crosslinked thin films with only UV light illumination. During the process, heating or any additional chemicals are not required. Moreover, the byproducts can be effectively removed by post-exposure immersion in a solvent. Because the major component of these systems is a poly(ethylene glycol)-based unit that can exhibit biocompatibility, the system can be used for fabricating organic thin film that can be used in a 2D extracellular matrix [35]. To facilitate cell adhesion, a certain amount of fGelMA, which is known to possess cell-adhesive ligands [50], was mixed with MGN or MDN. The concentration of the fGelMA was varied from 10 wt% to 100 wt%, relative to the amount of copolymer. In addition, LAP was added to the system to radically activate the methacryloyl group of the fGelMA to form the crosslinked network. Upon UV light illumination (500 mJ/cm^2^) of the mixed system, the radical-based crosslinking of the fGelMA chains and the crosslinking of the MGN or MDN copolymer chains occurred simultaneously. In the photocrosslinked fGelMA/MGN1, fGelMA/MGN5, fGelMA/MDN1, and fGelMA/MDN5 thin films, the lysine in fGelMA was utilized to label FITC to confirm the successful immobilization of fGelMA in the thin films. All the samples show green fluorescence in the CLSM image (Figure S9a–d), and more importantly, an increase in the mean fluorescence intensity was observed for all types of thin films (Figure S9e). These results confirm that the amount of fGelMA, the component that imparts the surface-active property, can be effectively controlled in thin films. It should be noted that even when fGelMA was mixed with the same amount of MGN or MDN (100 wt%), the thin films were found to be well-defined without any significant dewetting or phase separation. This was not the case for the ternary systems consisting of the same monomeric units and fGelMA [35], thereby elucidating the benefits of the single-component system over the multi-component system.

**Figure 9:**
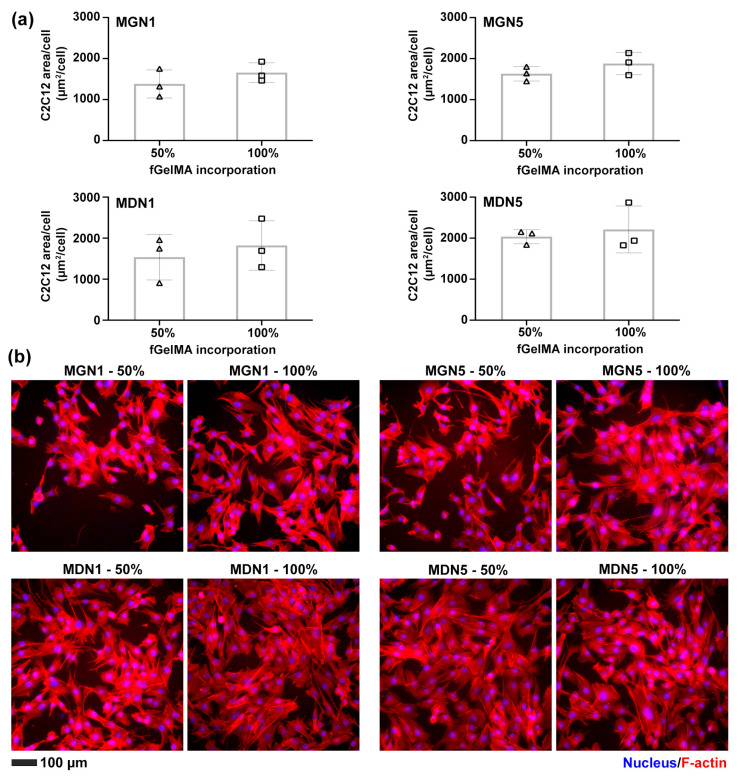
(**a**) Quantification of the C2C12 myoblast adhesion to the MGN and MDN thin films with the extent of fGelMA incorporation being varied from 50 wt% to 100 wt%, and (**b**) corresponding images of C2C12 myoblasts with F-actin and nuclei stained. Scale bar = 100 µm. Mean ± SD, n = 3, no significant difference after Student’s *t*-test (α = 0.05).

To assess the functionality of the fGelMA-incorporated thin films, cultures of C2C12 myoblasts were initiated. After 24 h, the cells were fixed, stained with phalloidin, and counterstained with Hoechst 33342. As shown in Figure 9a, no statistical difference was observed between 50 wt% and 100 wt% incorporation of fGelMA. In general, the phalloidin-stained areas on MGN1 or MDN1 are smaller than those on MGN5 or MDN5. The micrographs in the bottom rows (MDN terpolymers) show higher areas of phalloidin staining than those in the top rows (MGN terpolymers), as evidenced by Figure 9b. Interestingly, our previous work on the ternary blend of MN/MD or MN/MG with fGelMA showed invariably approximately 500 µm^2^/cells [35], while the current body of investigation shows 1400–2200 µm^2^/cells, depending on the terpolymer systems. These results further confirm that the terpolymer systems, as opposed to the ternary blend system, were able to improve the display of cell adhesive ligands by preserving such functional groups.

## 4. Conclusions

In this study, the synthesis and characterization of photocrosslinkable reactive copolymers and processes to form functionalizable thin films on various substrates in a single-component or a double-component composite with fGelMA are examined. The MGN and MDN terpolymers with varying compositions were successfully synthesized by RAFT polymerization in a controlled manner. Thin films of terpolymers were readily deposited on both organic and inorganic substrates by spin-coating their solution in ethanol. The UV light illumination led to effective crosslinking without heating or any additives. In the crosslinking reaction, DTC showed higher reactivity compared to the epoxide because MDN was easily crosslinked at ~100 mJ/cm^2^, whereas MGN required energy of >300 mJ/cm^2^. Moreover, the crosslinking of MDN was not affected by its composition; however, the composition of MGN was found to be significant in determining the sensitivity of the photocrosslinking reaction. After the crosslinking, the presented reactive functional groups in the thin films, i.e., primary amine, DTC (or epoxide), and thiol, were assessed for addition reactions with the isothiocyanate of FITC, the primary amine of the TAMRA amine or the lysine in BSA-FITC, and the maleimide of the Cy5 maleimide. All the thin films showed successful functionalization reactions, regardless of the composition. The composition affected the intensity of the fluorescence signal, suggesting the feasibility of controlling the amounts of reactive groups in the thin films. Furthermore, in one MDN5 thin film, both the addition reaction of the DTC with BSA-FITC and the thiol-ene click reaction with Cy5 maleimide were successful regardless of the reaction order, highlighting the orthogonality of the presented functionalities in the thin film. The thin films were effective in controlling the surface wettability through the post-crosslinking modification reaction with different primary amine compounds under ambient conditions. They were also beneficial in attaining an active surface for conferring cell adhesion in aqueous media by mixing with fGelMA, which resulted in a composite thin film without significant phase separation that further resulted in 3–4 times improvement of cell adhesion when compared to the thin films consisting of multiple polymers. These results underscore the significance of our chemically designed, simple yet effective single-component copolymer coating systems, which offer readiness, mildness, substrate independence, and desirable surface reactivity. Furthermore, they provide chemical tunability and the capability to define multiple chemical functionalities orthogonally, and they are scalable and applicable in various fields such as surface and biological engineering.

## Figures and Tables

**Table 1:** Feed compositions for the synthesis, molecular weight, dispersity, and actual compositions of the copolymers.

Sample	*f*_PEGMEMA_	*f*_GMA_ or *f*_DTC_	*f*_NBOCAEMA_	M_n_ (kg/mol)	*Đ*	*F*_PEGMEMA_	*F*_GMA_ or *F*_DTC_	*F*_NBOCAEMA_
MGN1	0.700	0.200	0.100	17.7	1.30	0.690	0.207	0.103
MGN2	0.750	0.150	0.100	14.2	1.34	0.758	0.136	0.106
MGN3	0.700	0.150	0.150	17.4	1.29	0.714	0.143	0.143
MGN4	0.750	0.100	0.150	14.4	1.27	0.744	0.100	0.156
MGN5	0.700	0.100	0.200	13.1	1.26	0.694	0.118	0.188
MDN1	0.700	0.200	0.100	22.4	1.53	0.758	0.152	0.091
MDN2	0.750	0.150	0.100	19.0	1.41	0.788	0.118	0.094
MDN3	0.700	0.150	0.150	14.3	1.38	0.736	0.132	0.132
MDN4	0.750	0.100	0.150	20.0	1.34	0.775	0.078	0.147
MDN5	0.700	0.100	0.200	20.9	1.34	0.720	0.100	0.180

## Data Availability

The datasets generated and/or analyzed during the current study are available from the corresponding author on reasonable request.

## Supplementary Materials

The following supporting information can be downloaded at: https://www.mdpi.com/article/10.3390/polym16010044/s1, ^1^H NMR spectra and SEC chromatograms (Figures S1 and S2); Optical images of water contact angle measurements (Figures S3 and S8); Thickness and water contact angle data (Figure S4); Optical micrographs and CLSM images showing photopatterns (Figures S5 and S6); Mean fluorescence intensity (Figures S7 and S9e); CLSM images of fGelMA/MGN and fGelMA/MDN composite thin films (Figure S9).
