# Supplementary material for: Single-Component Hydrophilic Terpolymer Thin Film Systems for Imparting Surface Chemical Versatility on Various Substrates
Source: Polymers (Basel). 2023 Dec 21;16(1):44. doi: 10.3390/polym16010044 (PMC10780973; doi:10.3390/polym16010044)
Supplement: Supplementary file 1 [file polymers-16-00044-s001.zip › polymers-2758584-supplementary.pdf]

Supplementary Material

Single Component Hydrophilic Terpolymer Thin Film Systems for Imparting Surface Chemical Versatility on Various Substrates

Yun Hee Ko,[1] *Hai Ha Tran Nguyen,*[1,2] *Christopher R. Branstetter,*[3] *Soeun Park,*[1] *Jin-Kyun Lee,*[4,5] *Jaesung Yang,*[6,]* *Jangwook P. Jung,*[3,]* *and Myungwoong Kim*[1,]*

[1]Department of Chemistry and Chemical Engineering, Inha University, Incheon 22212, Republic of Korea.

[2]Department of Applied Chemistry, Reutlingen University, Alteburgstraße 150, Reutlingen 72762, Germany.

[3]Department of Biological Engineering, Louisiana State University, Baton Rouge, Louisiana 70803, United States

[4]Program in Environment and Polymer Engineering, Inha University, Incheon 22212, Republic of Korea

[5]Department of Polymer Science and Engineering, Inha University, Incheon 22212, Republic of Korea

[6]Department of Chemistry, Yonsei University, Wonju, Gangwon 26493, Republic of Korea.

*Corresponding authors: Myungwoong Kim (mkim233@inha.ac.kr); Jangwook P. Jung (jjung1@lsu.edu); Jaesung Yang (jaesung.yang@yonsei.ac.kr)

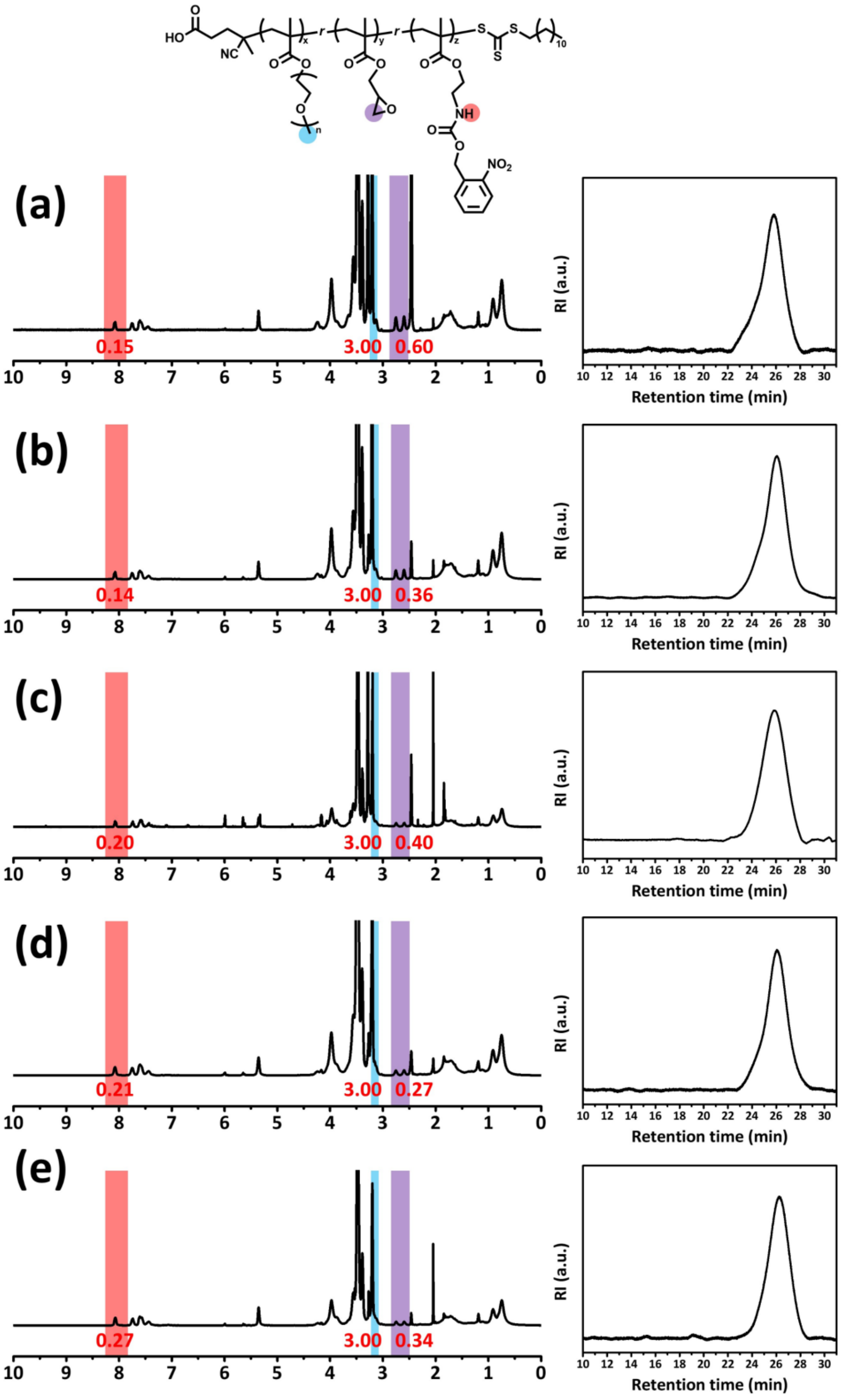

Figure S1. ^{1}H NMR spectra and SEC traces of the synthesized (a) MGN1, (b) MGN2, (c) MGN3, (d) MGN4, and (e) MGN5 copolymers.

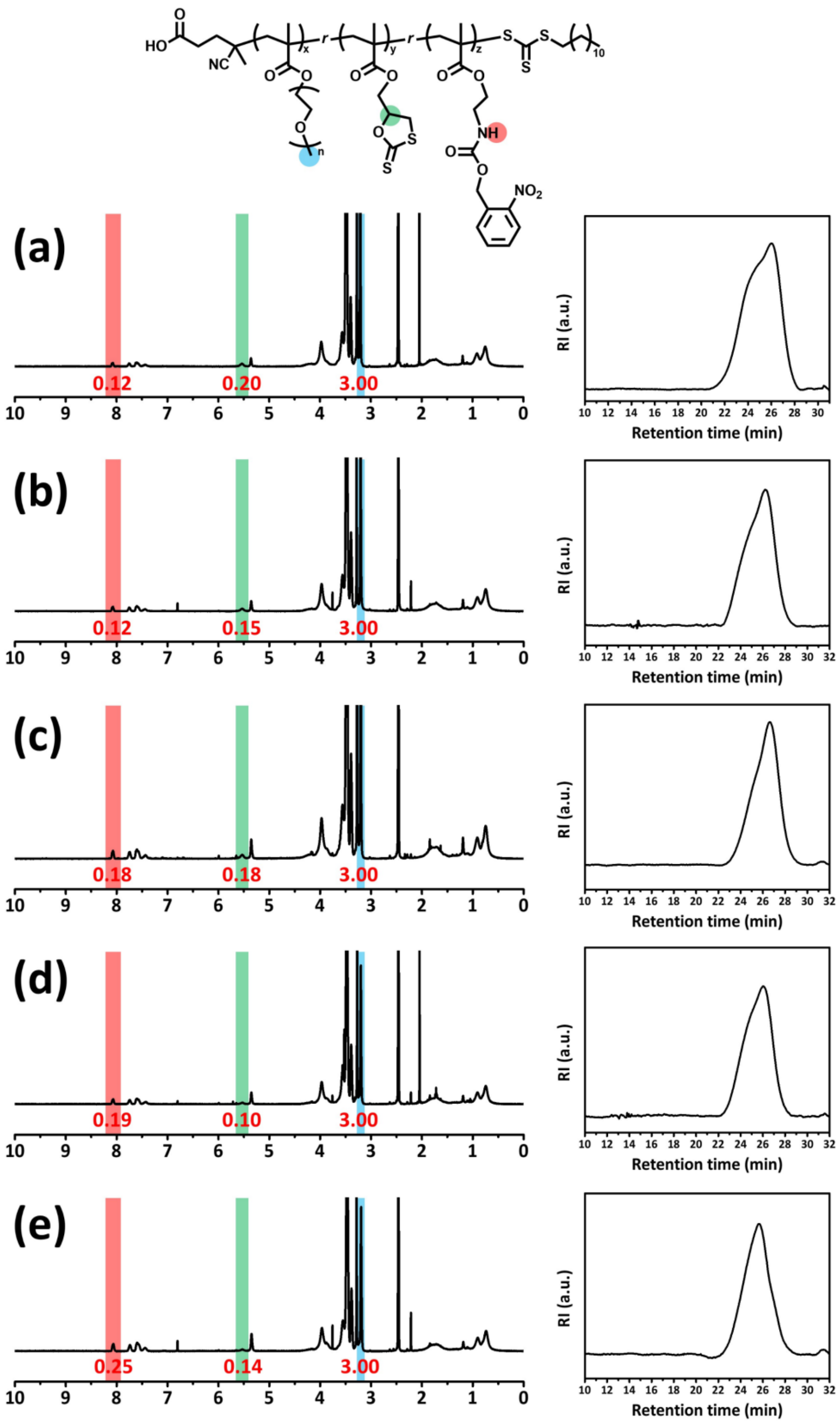

Figure S2. ^{1}H NMR spectra and SEC traces of the synthesized (a) MDN1, (b) MDN2, (c) MDN3, (d) MDN4, and (e) MDN5 copolymers.

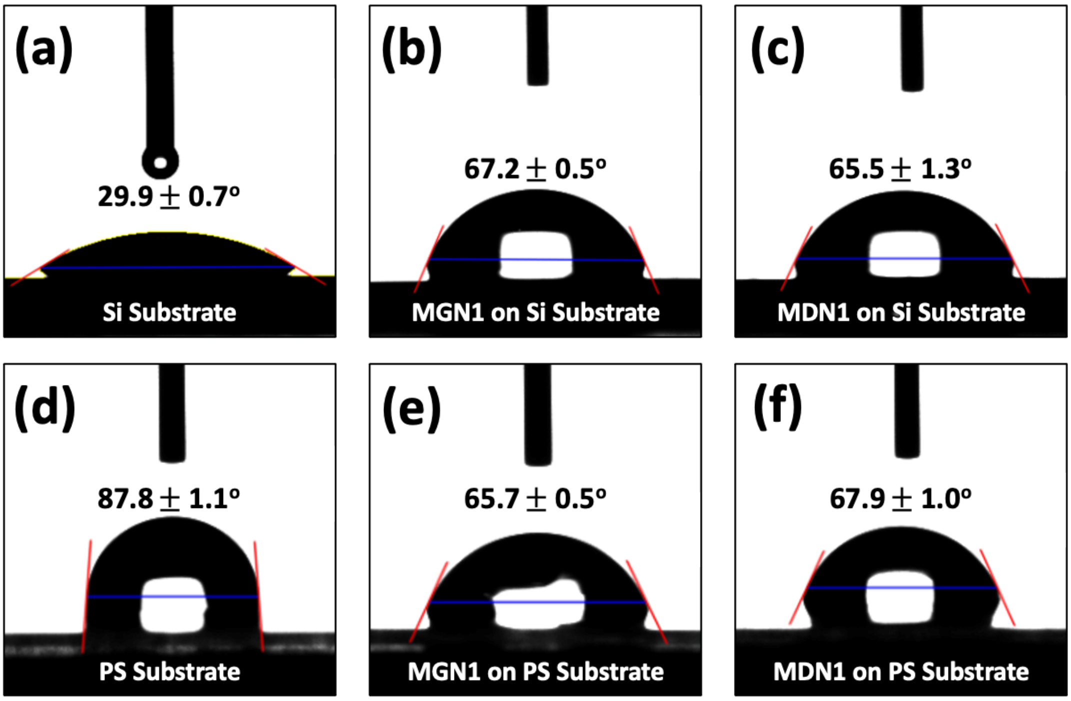

Figure S3. Representative optical images of water sessile drops on (a) bare silicon, (b) MGN1-coated on silicon, (c) MDN1-coated on silicon, (d) bare PS, (e) MGN1-coated on PS, and (f) MDN1-coated on PS substrates.

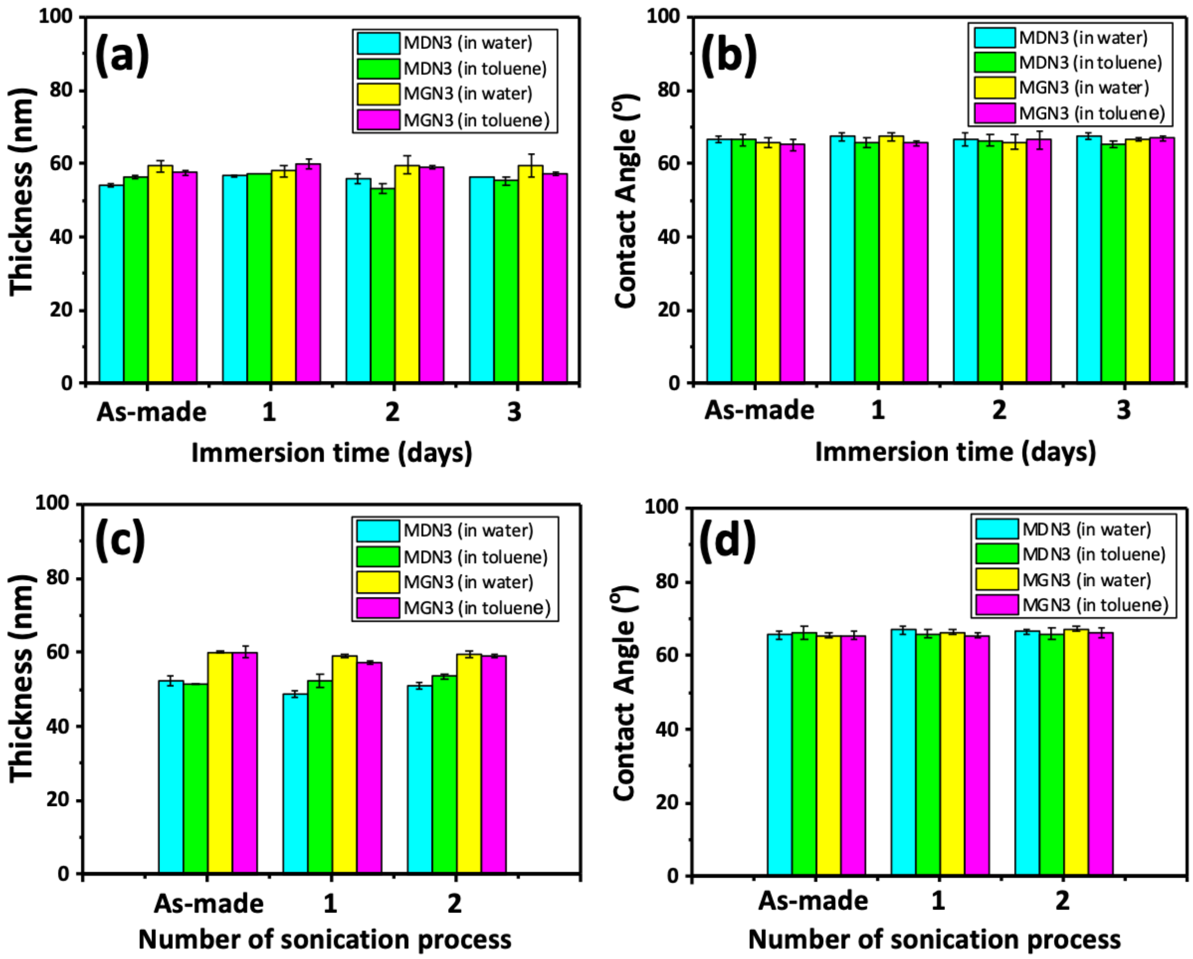

Figure S4. Thickness and water contact angle of MDN3 and MGN3 thin films (a and b) immersed in DI water or toluene and (c and d) sonicated in DI water or toluene.

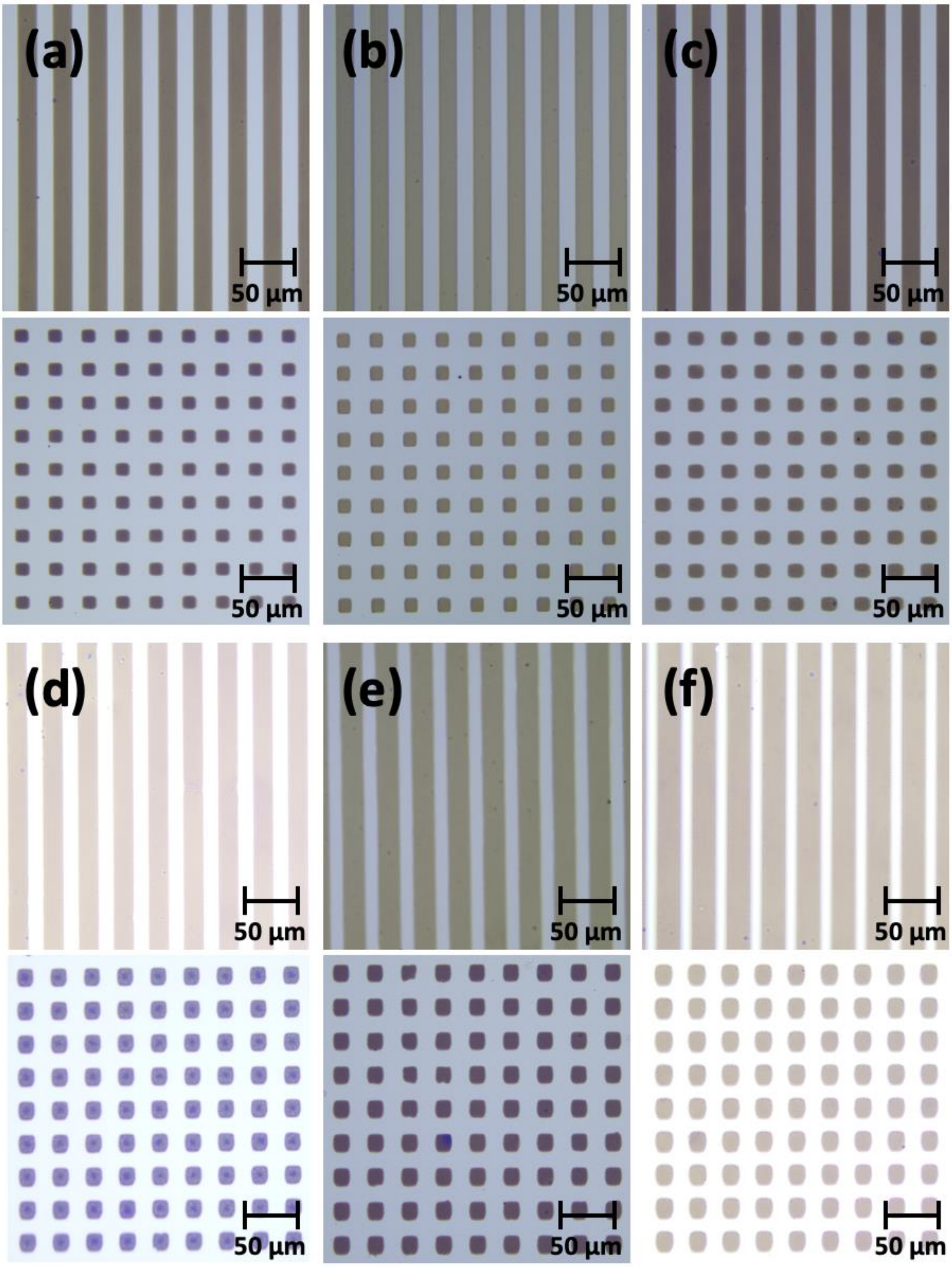

Figure S5. Optical micrographs showing line/space and square patterns fabricated with (a) MGN2, (b) MGN3, (c) MGN4, (d) MDN2, (e) MDN3, and (f) MDN4 copolymers (scale bar = 50 µm).

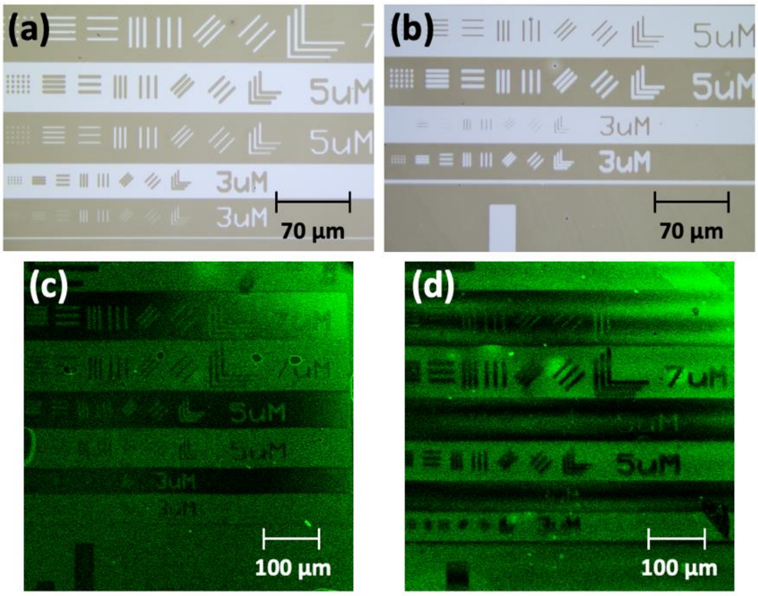

Figure S6. Optical micrographs of the photopatterns fabricated with (a) MDN3 and (b) MGN3, and fluorescence micrographs of FITC-labelled photopatterns of (c) MDN3 and (d) MGN3.

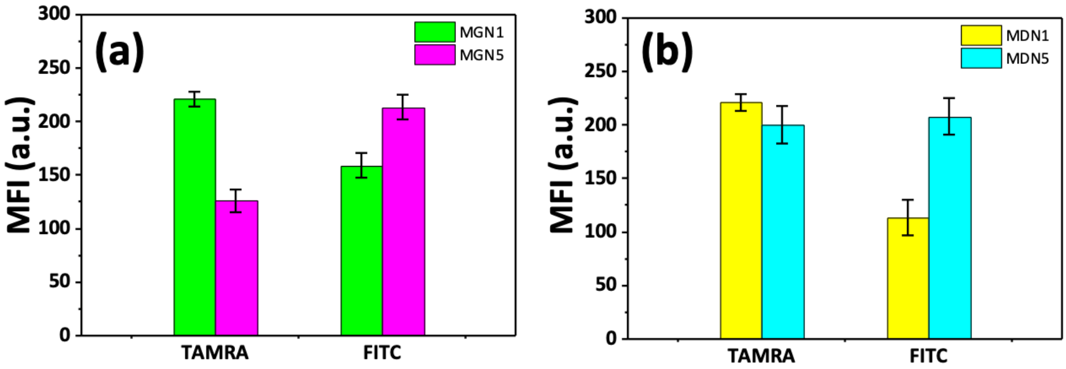

Figure S7. Mean fluorescence intensity (MFI) measured from TAMRA- and FITC-incorporated photopatterns fabricated with (a) MGN1 and MGN5, and (b) MDN1 and MDN5 copolymers.

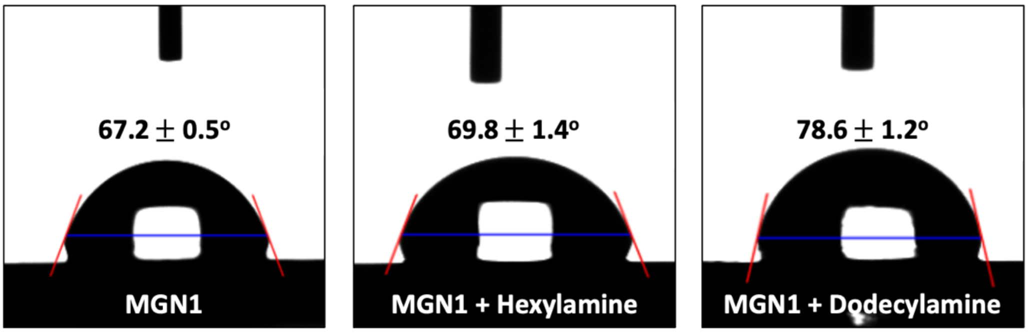

Figure S8. Optical images showing water sessile drops on the surfaces of unmodified, hexylamine-, and dodecylamine-immobilized MGN1 thin films.

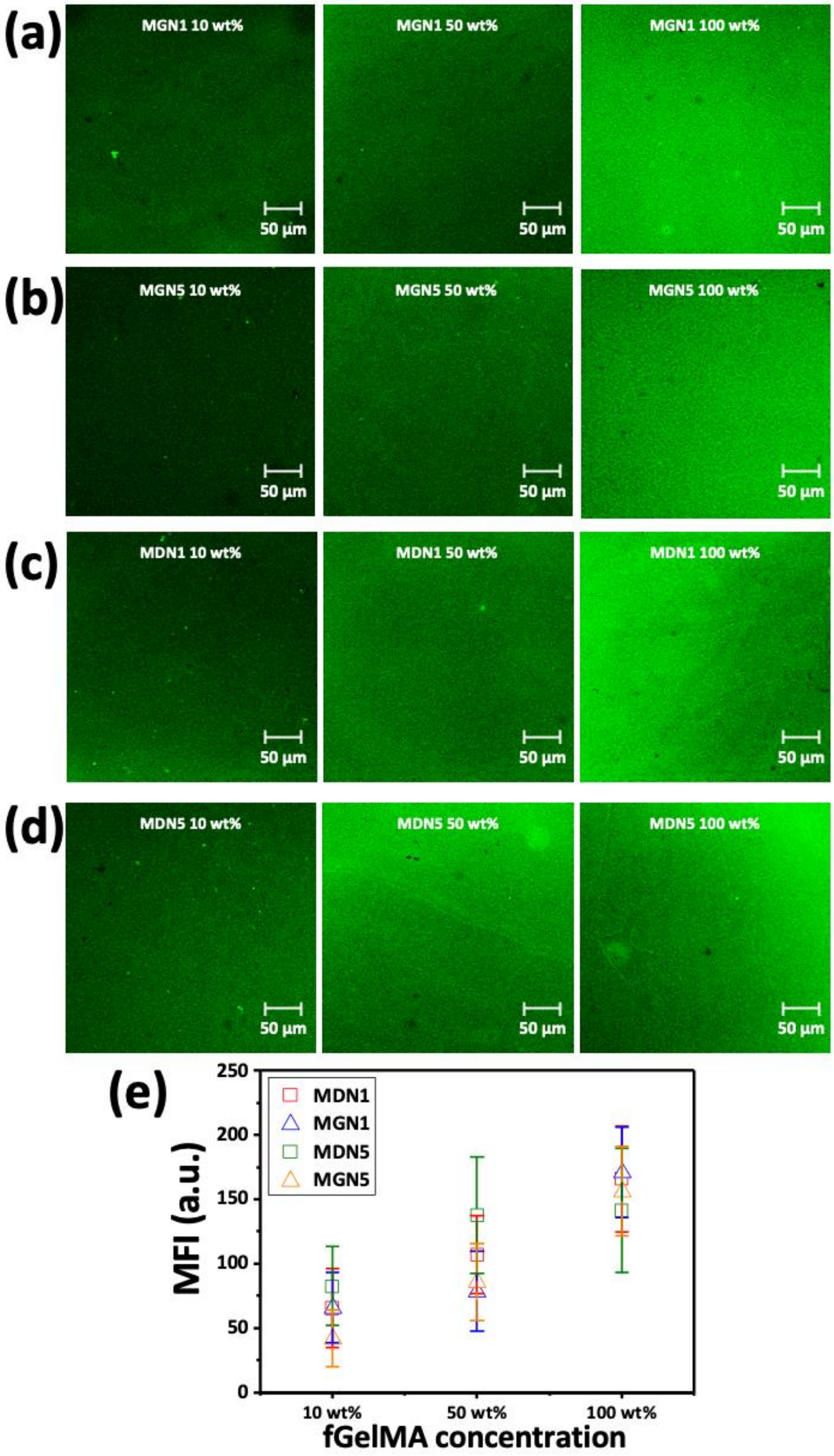

Figure S9. CLSM images showing the fluorescence signal of FITC from FITC-labelled (a) fGelMA/MGN1, (b) fGelMA/MGN5, (c) fGelMA/MDN1, (d) fGelMA/MDN5, and (e) MFI values estimated from the images of Figure **S9a-S9d**.
